# Ultrasound manifestations of enchondroma protuberans: Two case reports

**DOI:** 10.1097/MD.0000000000011161

**Published:** 2018-06-22

**Authors:** Xi Xiang, Shan Cheng, Yu-jia Yang, Li Qiu

**Affiliations:** Department of Ultrasound, West China Hospital of Sichuan University, Chengdu, Sichuan, China.

**Keywords:** bone tumor, diagnosis, enchondroma protuberans, imaging, ultrasound

## Abstract

**Rationale:**

We report two rare cases of enchondroma protuberans originating from phalanxes.

**Patient concerns:**

The patients visited doctors for a palpable mass in their phalanx without any pain or discomfort.

**Diagnoses:**

Biopsy is the gold standard for the diagnosis of enchondroma protuberans. Radiographs usually provide important imaging information, while studied on the ultrasound manifestation of enchondroma protuberans are still limited. In our cases, significant information about ultrasound manifestation of enchondroma protuberans were presented. Sonographic examination of enchondroma protuberans revealed a hypoechoic mass located in and beyond the medullary cavity of bone through the interrupted bone cortex, and blood flow signals were usually not abundant.

**Interventions:**

Patients were subsequently referred for surgical removal and the masses were confirmed by following pathological examination.

**Outcomes:**

After surgery, the patients recovered well with no relapse within 2 years.

**Lessons:**

Enchondroma protuberans is a rare form of benign enchondroma. Enchondroma protuberans can present as an intramedullary hypoechoic mass extending to the surrounding soft tissue via the discontinuous cortex line on ultrasound. Ultrasound can provide important information for the diagnosis of enchondroma protuberans.

## 1 Introduction

Enchondromas are benign, cartilaginous tumors that are the most common (90%) benign bone tumors affecting the hand.^[1–3]^ They most commonly affect the long bones of the hand, followed by the flat bones, and predominantly occur in the tubular bones.^[4]^ Enchondroma protuberans is a rare type of enchondroma, defined as an exophytic enchondroma of bones because of its location; the phalanges or metacarpal bones are the most common location involved.^[5,6]^ The main difference between enchondroma protuberans and the conventional enchondroma is the protruded mass.^[7]^ Therefore, the presence of an exophytic mass can clearly distinguish enchondroma protuberans from conventional enchondroma.^[6]^ The clinical symptoms of conventional enchondroma are subtler than those of enchondroma protuberans. Patients with conventional enchondromas can be asymptomatic, or can experience pain, and sometimes a pathological fracture, whereas enchondroma protuberans usually presents as a palpable mass in the phalanx. Radiographic imaging studies, such as X-ray, computed tomography (CT), and magnetic resonance imaging (MRI) on enchondroma protuberans are available, but those regarding ultrasound findings remain scarce. We retrospectively analyzed the clinical and sonographic features of 2 cases with exophytic masses that were confirmed pathologically as enchondromas in this article.

## 2 Case presentation

The 2 cases included were sonographically evaluated by using the iU22 scanner (Philips Healthcare, Andover, MA) equipped with a 12-5 MHz linear-array transducer. Both of the cases underwent mass resection and pathological examination, and recovered well after surgery with no relapse within 2 years. No extra intervention was involved in the 2 cases. Adverse and unanticipated events did not occur during the diagnosis and treatment progress. Complete clinical, ultrasonic, and pathological data were available. No family history or genetic history was found.

### 2.1 Case 1

**Figure 1:**
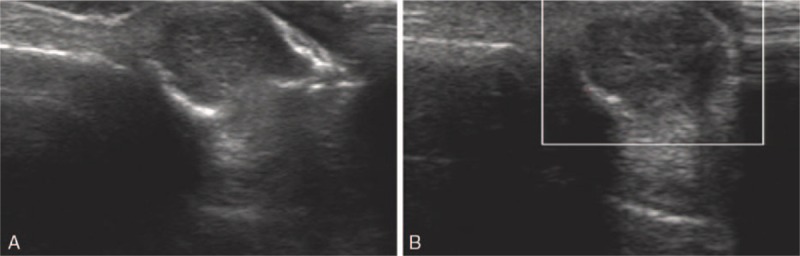
Case 1. (A) Two-dimensional ultrasound showed a hypoechoic mass located in and beyond the medullary cavity of the phalanx. (B) Doppler ultrasound showed no blood signal in the mass.

A 48-year-old woman noticed a palpable mass in the distal phalanx of her left ring finger with a medical history of enchondroma in the same location. Ultrasound examination revealed an exophytic hypoechoic mass that was connected to the medullary cavity through the interrupted bone cortex. No obvious blood signals or calcification were found in this area (Fig. 1). It is difficult to characterize or classify the mass by traditional ultrasound. And a mass located close to the bone was described in our ultrasound diagnostic report.

### 2.2 Case 2

**Figure 2:**
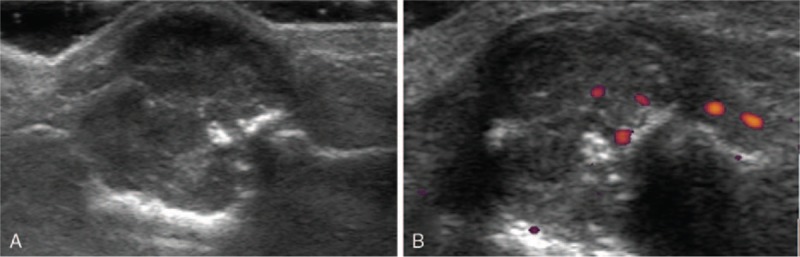
Case 2. (A) Two-dimensional ultrasound showed punctuate or patchy calcification in the mass, and discontinuous bone cortex. (B) Point-like blood signal presented in Doppler ultrasound.

A 25-year-old woman underwent ultrasound examination for a palpable mass in the middle phalanx of her left ring finger. A hypoechoic mass of 20 mm diameter was found. The boundary of the mass was unclear and punctuate or patchy hyperechoic calcification was present in the mass. The bone cortex was involved, with a broken continuity. In addition, a point-like blood signal was observed on switching to Doppler mode (Fig. 2). Notably, the pathology report showed active proliferation in the tumor cells. A mass with bone erosion was diagnosed.

## 3 Discussion

Enchondroma protuberans is usually caused by enchondroma expansion through the bone cortex leading to a single lesion in the phalanx. However, it has a much lower morbidity than conventional enchondroma. Enchondroma protuberans is a rare, benign chondromatous tumor that arises in the medullary canal, forming an exophytic mass in the surrounding soft tissue.^[8]^ Unlike osteochondroma, the cartilage of the enchondroma protuberan was not a cap over normal trabecular bone, but extended outward from an intramedullary locus.^[9]^ A few small tumors or those with slow growth require close monitoring instead of surgery. Those with obvious symptoms, rapid growth, or pathologic fracture need timely surgery. The early detection of enchondroma protuberans is important.

There are no exact diagnostic criteria for enchondroma protuberans, and biopsy provides the most convincing evidence for diagnosis. Radiographs can provide important imaging information. Radiologically, enchondroma protuberans is presented as a well-defined intramedullary osteolytic lesion and may be accompanied by fine matricidal calcification, cortical expansion and cortical defect, and round well-defined soft tissue expansion.^[7]^ For example, an enchondroma protuberans in a rib may present an eccentrically located, calcified mass expanding the bone on X-ray, and the junction of bone and cartilage is almost intact. A technetium-99 M radioisotope bone scan demonstrates slightly diffuse increased uptake. A specimen radiograph may demonstrate dense confluent calcifications in both the medullary and extraosseous portions of the tumor.^[9]^ The typical presentation of enchondroma protuberans in MRI is a well-defined intramedullary lesion with low signal intensity in T1-W image and high signal intensity in T2-W and short time inversion recovery sequences accompanied by cortical expansion and cortical defect.^[7]^

Ultrasound shows great promise in musculoskeletal system imaging and can provide significant information in the diagnosis and detection of diseases. However, reports on ultrasound findings of enchondroma protuberans are limited. Therefore, sonographic appearances of enchondroma protuberans were analyzed and discussed in this article.

The 2 cases with exophytic masses confirmed on pathological examination as enchondromas shared some similarities, for example, the lesions were solitary and closely related to the phalanges, which met the predilection sites of enchondroma protuberans. Sonographic appearance had certain characteristics (Table 1). In both cases, the tumor appeared as a hypoechoic mass occurring in both the medullary cavity and surrounding soft tissue, with a discontinuous bone cortex. Masses extended beyond the medullary cavity through the interrupted bone cortex. The internal echo of both lesions was relatively homogeneous and no liquefaction was observed. On the contrary, there were some differences in sonographic appearance between the 2 cases. No calcification was present in case 1, while punctuate and patchy hyperechoic calcification was seen in case 2. An early study reported that enchondroma could show a well-defined lucency with punctuate calcification, and endosteal erosion and cortical thinning may be observed particularly in the short tubular bones,^[10]^ which was in agreement with our findings. Moreover, blood supply of the tumors differed in the two cases. Generally, blood supply of benign bone tumors is always minimal and scattered, and enchondroma is no exception. In Doppler mode, blood signal could hardly be observed in case 1, while punctate signal was observed in case 2. In addition, biopsy of case 2 showed an active proliferation of a part of the tumor cells. This may indicate a relationship between blood supply and degree of tumor cell activity. A preliminary conclusion about ultrasound manifestation in enchondroma protuberans is achieved, that is, a hypoechoic mass appears in and beyond medullary cavity with an interrupted hyperechoic cortical line, while punctuate calcification and blood supply may exist. When the tumor cells proliferate actively, punctate and linear blood signal may be observed on ultrasound.

**Table 1:**
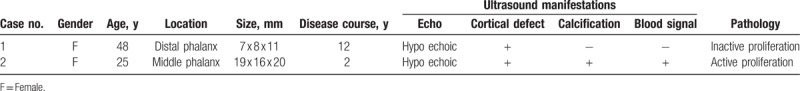
Clinical, ultrasonic, and pathological findings of enchondroma protuberans.

Cartilage tumor is a mass closely related to bone with homogeneous echo and less blood supply, including osteochondroma, periosteal chondroma, and enchondroma. Osteochondroma is continuous with the bone, and typically has a cartilaginous cap outside the lesion and does not contain cartilaginous tissue in its base or in the medullary cavity,^[2]^ whereas enchondroma, including enchondroma protuberans, does not have a cartilaginous cap. Periosteal chondroma is a benign cartilaginous tumor of periosteal origin, and usually located beneath the periosteum and has no connections to the medullary cavity.^[2,8,11]^ The absence of cartilaginous cap and underlying trabecular bone differentiates enchondroma from osteochondroma and delineation of contiguous intramedullary involvement can be a strong indicator to differentiate it from periosteal chondroma.^[7]^ Chondrosarcoma must be considered when distinguishing the properties of the lesion, especially in cases seen in costal bones, and more attention should be paid to low-grade chondrosarcoma.^[12]^ Most of the cartilage tumors present with characteristic features on imaging. However, it remains difficult to differentiate enchondroma and low-grade chondrosarcoma. Clinical symptoms, such as increasing size and onset of pain, may be of some help in diagnosis.

Despite the fact that ultrasound can provide important information for the diagnosis of enchondroma protuberans, it is still not as suitable as radiographs for the assessment of bone. In addition, radiographs cannot be replaced with ultrasound in the aspect of the diagnosis of enchondroma protuberans.

## 4 Conclusion

We report 2 patients of palpable masses on the phalanx, and we suspect that enchondroma protuberans can present as an intramedullary hypoechoic mass extending to the surrounding soft tissue via the discontinuous cortex line. Punctuate or patchy calcification and blood supply may occur in some cases. Appearance of blood signal may indicate tumor cell activity. Further study with a large sample size is needed to confirm it. Although ultrasound manifestation of enchondroma protuberans is atypical, awareness of enchondroma protuberans should be present when an exophytic mass originating in the medullary cavity is found on sonography.

## 5 Method

This is a case report. And it is not necessary to obtain an ethics committee or institutional review board approval. Otherwise, written informed consents were signed by the 2 patients.

## Author contributions

**Data curation:** Xi Xiang.

**Funding acquisition:** Li Qiu.

**Methodology:** Xi Xiang, Li Qiu.

**Resources:** Li Qiu.

**Software:** Xi Xiang.

**Writing – original draft:** Xi Xiang.

**Writing – review & editing:** Shan Cheng, Yujia Yang.
